# Effects of cell culture time and cytokines on migration of dental pulp stem cell‐derived chondrogenic cells in collagen hydrogels

**DOI:** 10.14814/phy2.70063

**Published:** 2024-09-26

**Authors:** Li Yao, Nikol Flynn, Pranita Kaphle

**Affiliations:** ^1^ Department of Biological Sciences Wichita State University Wichita Kansas USA

**Keywords:** cell migration, collagen, dental pulp stem cell, differentiation, nucleus pulposus

## Abstract

The transplantation of collagen hydrogels encapsulating human dental pulp stem cell (DPSC)‐derived chondrogenic cells is potentially a novel approach for the regeneration of degenerated nucleus pulposus (NP) and cartilage. Grafted cell migration allows cells to disperse in the hydrogels and the treated tissue from the grafted location. We previously reported the cell migration in type I and type II hydrogels. It is important to explore further how cell culture time affect the cell motility. In this study, we observed the decreased motility of DPSC‐derived chondrogenic cells after culturing for 2 weeks compared with cells cultured for 2 days in these gels. The Alamarblue assay showed the cell proliferation during the two‐week cell culture period. The findings suggest that the transitions of cell motility and proliferation during the longer culture time. The result indicates that the early culture stage is an optimal time for cell transplantation. In a degenerated disc, the expression of IL‐1β and TNFα increased significantly compared with healthy tissue and therefore may affect grafted cell migration. The incorporation of IL‐1β and TNFα into the collagen hydrogels decreased cell motility. The study indicates that the control of IL‐1β and TNFα production may help to maintain cell motility after transplantation.

## Graphical abstract

The migration of DPSCs‐derived chondrogenic cells in collagen gel after transplantation into the nucleus pulposus. TNF‐α and IL1β inhibit cell migration in collagen gels.

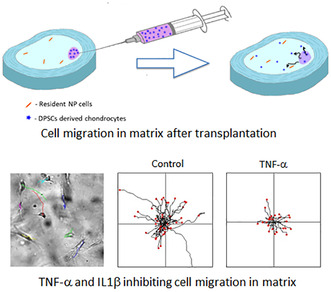

## 1 INTRODUCTION

Type II collagen is the major extracellular matrix component in nucleus pulposus (NP). Degenerated discs lose their matrix type II collagen and proteoglycans because of the elevated activity of metalloproteinases and inflammatory factors (Johnson et al., 2015). In addition, the altered microenvironment results in cell apoptosis and therefore deteriorates the degeneration process. Stem cell therapy is an emerging strategy for NP regeneration because the grafted cells may be able to replace lost native cells. Stem cells can be injected directly into the native tissue or delivered by hydrogel encapsulating the stem cells (Kumar et al., 2016; Wang et al., 2019; Xing et al., 2021). Collagen hydrogel that mimics the mechanical properties and microstructure of the native tissue may help to improve the integration of grafted cells within the NP tissue (Priyadarshani, Li, & Yao, 2016). In previous studies, type II collagen and hyaluronan (HA) formed a hydrogel that functioned as a matrix to support mesenchymal stem cells (MSCs), bovine NP cells, and human NP cell growth (Calderon et al., 2010; Collin et al., 2011; Priyadarshani, Li, Yang, & Yao, 2016).

Human dental pulp stem cells (DPSCs) are attractive for NP and cartilage generation. These cells are easily accessible and have demonstrated similar differentiation capability as other MSCs (Cui et al., 2021; Huang et al., 2009). The transplantation of DPSC‐derived chondrogenic cells encapsulated in type II collagen hydrogels mimicking the native NP tissue and cartilage is a potentially novel approach to regenerating these degenerated tissues. The grafted cell migration allows cells to disperse from the grafted location and integrate into the treated tissue. Exploring cell motility in the collagen matrices will help to understand the cellular process after transplantation. In a previous study, we reported the differentiation of DPSCs toward chondrogenic cells and cell cultures in type I and type II collagen hydrogels (Yao & Flynn, 2018). We also reported the differentiated cell migration in type I and type II collagen gels. It is important to explore further how cell culture time affect the cell motility in the gels. In this study, DPSC‐derived chondrogenic cells were grown in collagen hydrogels for 2 weeks, and their motility was studied at early (2 days) and late stages (2 weeks) of the cell culture. Results provide clues for the capability of cell migration at different cell culture stages. Previous study showed that the rigidity of cell culture surface affected the cell migration speed (Ulrich et al., 2009). However, the migration of DPSC‐derived chondrogenic cells on different rigidity surfaces has not been reported. Particularly, it is interesting to compare the cell migration in the gel and on collagen coated culture dish representing cartilage surface. This study investigated the cell migration inside type II collagen gel and on a stiff cell culture dish coated with type II collagen.

In degenerated cartilage and disc, the expression of chmokines such as IL‐1β and TNFα increased significantly compared with healthy cartilage and NP tissue (Johnson et al., 2015; Risbud & Shapiro, 2014). It has been reported that IL‐1β and TNFα inhibit the migration of chondrogenic progenitor cells from non‐fibrillated osteoarthritic cartilage (Joos et al., 2013). In the treatment of degenerated NP, the grafted DPSC‐derived chondrogenic cells will experience an increased amount of IL‐1β and TNFα in the tissue. However, it is not clear how chemokines affect the motility of DPSC‐derived chondrogenic cells in collagen hydrogels. In this study, IL‐1β and TNFα were loaded into the collagen hydrogel, and then the effect of these factors on cell migration was tested.

## 2 MATERIALS AND METHODS

### 2.1 Study of cell migration in type I and type II collagen hydrogels at early and late cell culture stages

Chondrogenic differentiation from DPSCs (PT‐5025, Lonza, Walkersville, MD) was performed as the method that we previously reported (Yao & Flynn, 2018). The DPSC cell pellet was generated by centrifuging 250,000 cells, and the cell pellet differentiation was induced with high‐glucose Dulbecco's Modified Eagle Medium (DMEM) containing 10 ng/mL TGF‐β1 (100–21, PeproTech US, Cranbury, NJ), 50 g/mL ascorbate‐2‐phosphate, 0.1 M dexamethasone, 100 g/mL sodium pyruvate, 40 g/mL proline, 50 mg/mL ITS premix (I3146‐5ML, Sigma, St. Louis, MO), and 1% penicillin–streptomycin. After culturing for 2–3 weeks, the cell pellets were dissociated with collagenase type I (17,018,029, Thermo Fisher Scientific), which was prepared with a cell culture medium containing 10% fetal bovine serum (FBS), 0.15% collagenase type I, and 2 mM calcium chloride (CaCl_2_). The dissociated cells were grown on a cell culture dish for 6 days before seeding in collagen hydrogels to study the cell migration.

To study the cell migration in the collagen hydrogels, 400 μL type I collagen (5 mg/mL, extracted from bovine Achilles tendon) or type II collagen (5 mg/mL, extracted from bovine cartilage) solution was transferred into the wells of a 48‐well plate. The collagen type I and type II were extracted in the lab. The collagen solution was neutralized in the wells using 1 M sodium hydroxide solution and 10× phosphate‐buffered saline (PBS). The dissociated DPSC‐derived chondrogenic cells (40,000) were mixed into the collagen gel. The differentiation cell culture medium was used to maintain cell growth. The cell migration was recorded by time‐lapse microscopy after cell culturing in the gels for 2 days or 2 weeks.

### 2.2 Migration of cells in collagen hydrogels or on collagen‐coated dishes

In this study, we compared the migration of DPSC‐derived chondrogenic cells in type I or type II collagen gels, and the same cells on stiff culture dishes with or without a type II (5 mg/mL) collagen coating. The differentiated cell pellets were dissociated with collagenase type I, as described above. These dissociated cells were directly seeded into the collagen hydrogels or grown on the cell culture dish surface with or without collagen coating treatment. Cell migration was recorded after culturing for 1 day.

### 2.3 Cell migration recording using time‐lapse microscopy

The time‐lapse imaging of cell migration in the collagen hydrogels or on the cell culture dish was recorded using a Zeiss Axio Observer microscope, which was placed in a plastic chamber with temperature control at 37°C and a CO_2_ (5%) supply. Images were taken using a digital camera (AxioCam MRm Rev.3 with FireWire). Cell migration was recorded for 3 h, with one image every 5 min. At least three independent experiments were performed for each treatment condition. The cell migration velocity, accumulated cell migration distance, and Euclidean cell migration distance were quantified using NIH ImageJ software.

### 2.4 Proliferation of DPSC‐derived chondrogenic cells in hydrogels

The viability and proliferation of DPSC‐derived chondrogenic cells were measured using alamarBlue® assay (Pierce Biotechnology, Rockford, IL). This assay was performed after culturing was done for 2 days and 2 weeks. To perform the assay, the cell culture medium was removed from the cell culture well. The cell‐cultured medium containing 10% alamarBlue® reagent was added to the cell culture well. After incubation for 4 h in the incubator at (37°C and 5% CO_2_), the medium was transferred to a 96‐well plate, and the absorbance was measured using a microplate reader spectrophotometer (Synergy MX Monochromator‐Based Multi‐Mode Microplate Reader, Winooski, VT) at 570 nm and 600 nm. The percent reduction was calculated using the formula suggested by the manufacture.

### 2.5 Study of the effect of TNFα and IL‐1β on DPSC‐derived chondrogenic cells

To investigate the impact of TNFα (300‐01A, Thermo Fisher Scientific, Waltham, MA) and IL‐1β (200‐01B, Thermo Fisher Scientific, Waltham, MA) on the DPSC‐derived chondrogenic cell migration in the collagen gels, TNFα (20 ng/mL) and IL‐1β (2 ng/mL) were added to the neutralized type I collagen hydrogel. The DPSC‐derived chondrogenic cells (20,000) were then seeded into the prepared collagen gels. After culturing for 1 day, cell migration in the gels was recorded.

### 2.6 Statistics

The data is described as mean ± standard deviation. Statistical comparison of the means between experimental groups was analyzed using ANOVA (post‐hoc Bonferroni) by SPSS (SPSS Inc., Chicago, IL). The *p*‐value of 0.05 is considered to be statistically significant.

## 3 RESULTS

### 3.1 Decrease in MigrationVelocity of DPSC‐derived chondrogenic cells after culturing in collagen hydrogels for 2 weeks

**FIGURE 1:**
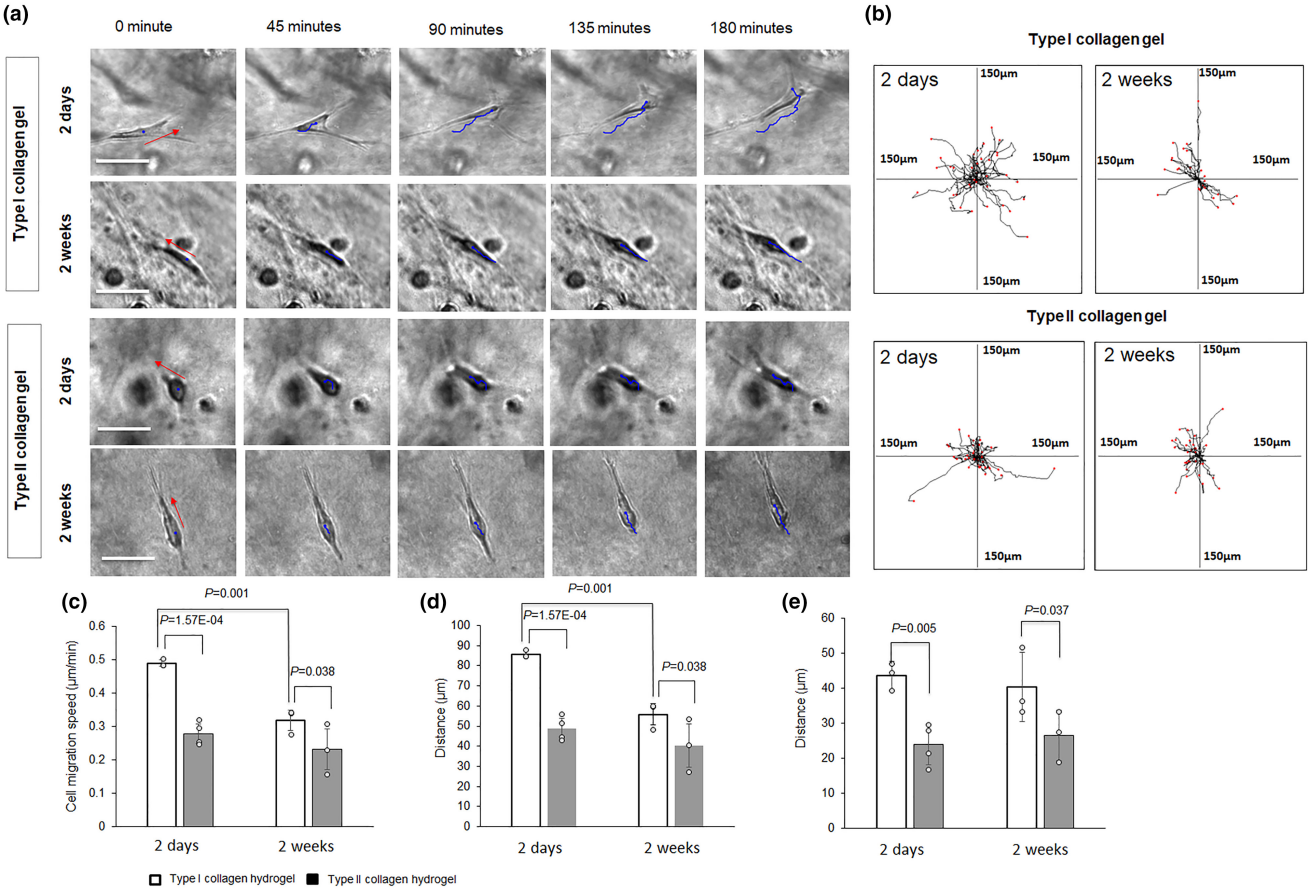
Migration of DPSC‐derived chondrogenic cells in collagen hydrogels at early and late cell culture stages. (a) DPSC‐derived chondrogenic cell migration in type I and type II collagen hydrogels cultured for 2 days and 2 weeks. Blue lines indicate the cell movement pathway. Scale bar: 50 μm. (b) Summary of migration pathways of cells from different experiments; center of each frame indicates migration initiation position of all cells under each experimental condition. (c) Cell migration speed. (d) Accumulated distance. (e) Euclidean distance.

Typical cells in type I or type II collagen hydrogels after culturing for 2 days or 2 weeks are shown in Figure 1a. The cell migration pathways are labeled with blue lines. Migrating cells were guided by cell‐leading processes. The cell migration pathways are indicated as superimposed lines in each frame of Figure 1b. The length of migration distance of the cells in collagen hydrogels decreased after culturing for 2 weeks, compared with cells cultured for 2 days.

The migration velocity was 0.48 ± 0.01 μm/min for DPSC‐derived chondrogenic cells cultured in type I collagen hydrogel for 2 days (Figure 1c). After culturing in type I collagen hydrogels for 2 weeks, the migration velocity was 0.39 ± 0.03 μm/min, which is significantly lower than that in the cell culture for 2 days (*p* < 0.01). The accumulated distance and Euclidean distance for cell migration in type I collagen hydrogels decreased after cell culturing for 2 weeks, compared with culturing for 2 days. The migration speed and distance of the cells were higher in type I collagen gels comparing with the cells in type II collagen gels after culturing for 2 days or 2 weeks.

### 3.2 Proliferation of DPSC‐derived chondrogenic cell in collagen hydrogel

**FIGURE 2:**
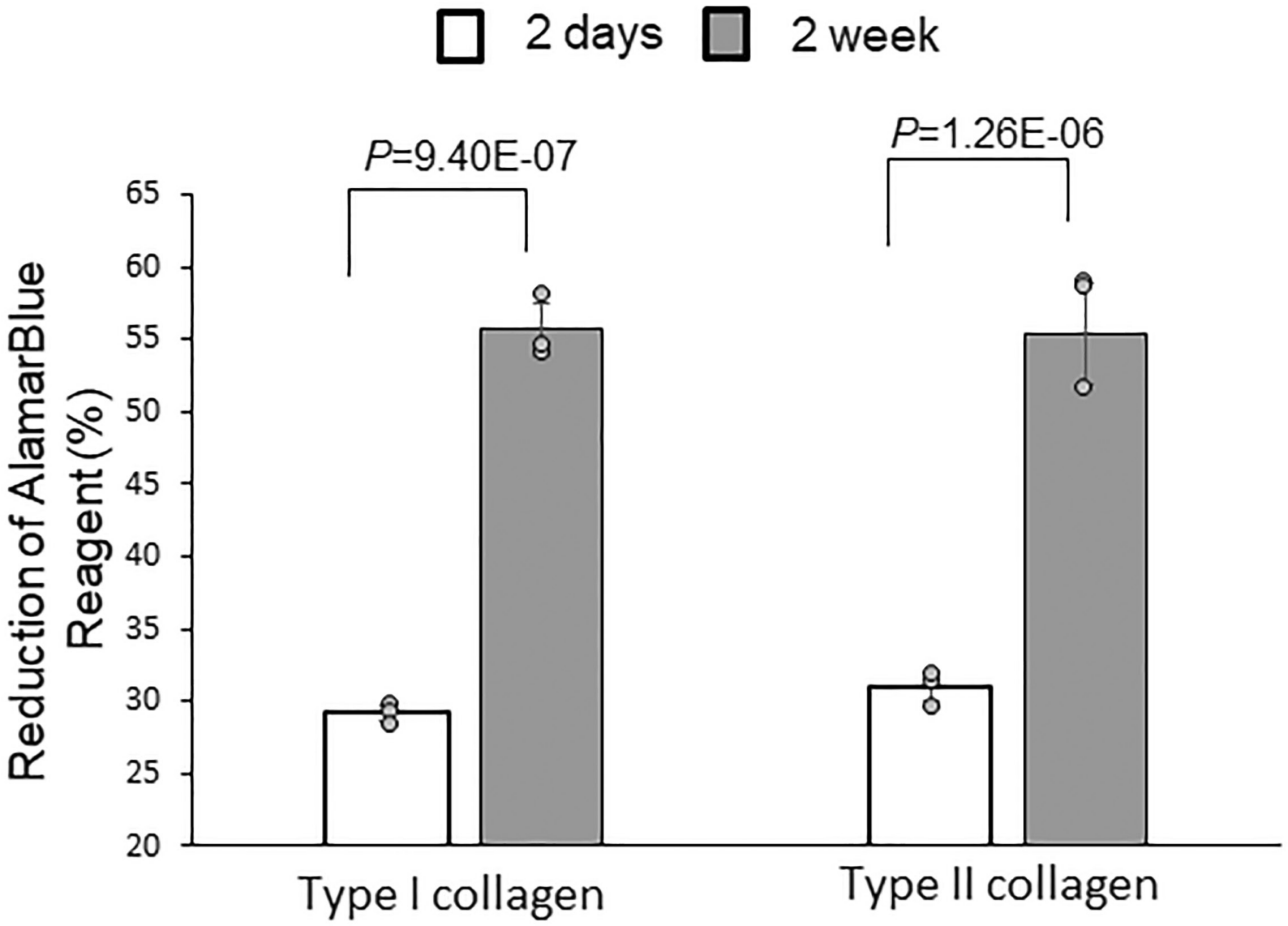
AlamarBlue® assay for DPSC‐derived chondrogenic cell growth in hydrogels for 2 weeks. Results show cell proliferation in type I and type II collagen hydrogels.

After the DPSC‐derived chondrogenic cells were cultured in type I and type II collagen hydrogels for 2 days, the reduction values of alamarBlue® reagent were 29.22 ± 0.54 and 31.04 ± 0.99, respectively (Figure 2). After culturing for 2 weeks, these alamarBlue® reagent reduction values were 55.65 ± 1.77 and 55.40 ± 3.45, respectively, which were significantly higher than the values at 2 days (*p* < 0.01). The increased reduction value of the alamarBlue® reagent indicates cell proliferation in the hydrogels.

### 3.3 Comparison of cell motility and cell body surface area for cells in collagen hydrogels and on cell culture dishes

**FIGURE 3:**
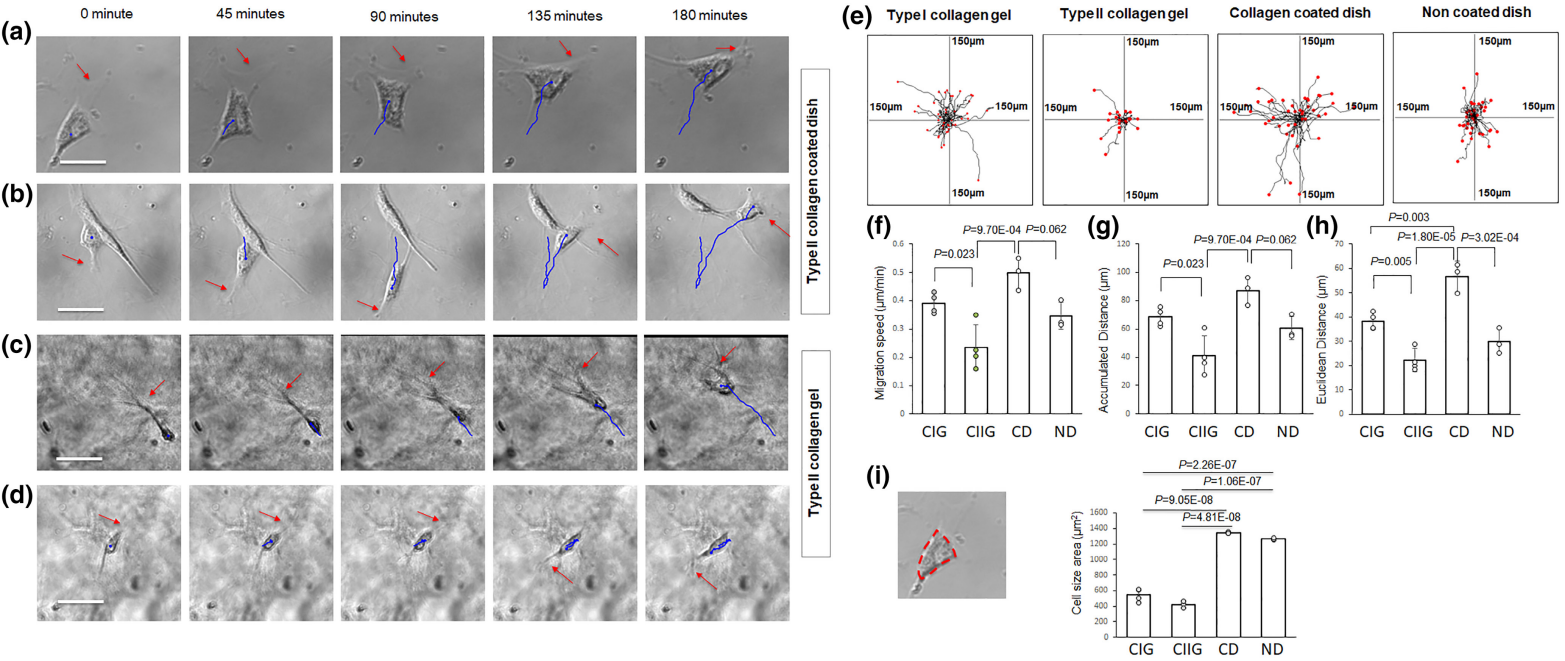
Quantitative results of DPSC‐derived chondrogenic cell migration tracking in type I, type II collagen hydrogels and type II collagen coated cell culture dish. (a–d) Representative cell migration on type II collagen‐coated dish (a, b) in type II collagen hydrogel (c, d). Blue color indicates cell migration pathways. Arrows indicate processes leading cell migration. Scale bar: 50 μm. (e) Summary of migration pathways of cells from different experiments; center of each frame indicates migration initiation position of all cells under each experimental condition. (f) Cell migration speed. (g) Accumulated distance. (h) Euclidean distance. (i) Cell body size quantification. CIG, type I collagen hydrogel; CIIG, type II collagen hydrogel; CD, type II collagen‐coated dish; ND, non‐coated cell culture dish.

To compare the cell migration in type II collagen hydrogel and on a type II collagen‐coated dish, the cells were directly seeded in the gel or on a stiff culture dish after dissociating the differentiated cell pellets. Sequential cell migration images for the cells in type II collagen gel (Figure 3a,b; see Videos S1 and S2) or on the type II collagen coated cell culture dish (Figure 3c,d; see Videos S3 and S4) show that the cell processes, labeled with red arrows, guided the cell migration. The extension of cell processes on the opposite side of the cell leading process reversed the cell migration direction (Figure 3b,d). The cell migration pathways in collagen gels or on cell culture dishes are shown as superimposed lines in Figure 3e. The cell migration velocity in type II collagen gel (0.23 ± 0.08 μm/min) was lower than that on the type II collagen‐coated dish (0.49 ± 0.05 μm/min, *p* < 0.01) (Figure 3f). The cell migration distance in type I collagen gel is longer than that in type II collagen gel (Figure 3g,h). The cell migration distance on type II collagen coated dish is longer than that in type II collagen gel (Figure 3g,h). The cell body area for the cells in the collagen gels or on the cell culture dishes was measured and quantified. The cell body surface area was outlined and measured by NIH ImageJ software. The cell body surface area in the hydrogels was smaller than that on the cell culture dishes (*p* < 0.01) (Figure 3i).

### 3.4 Decrease in cell migration velocity of DPSC‐derived chondrogenic cells in collagen hydrogel with IL‐1β and TNFα

**FIGURE 4:**
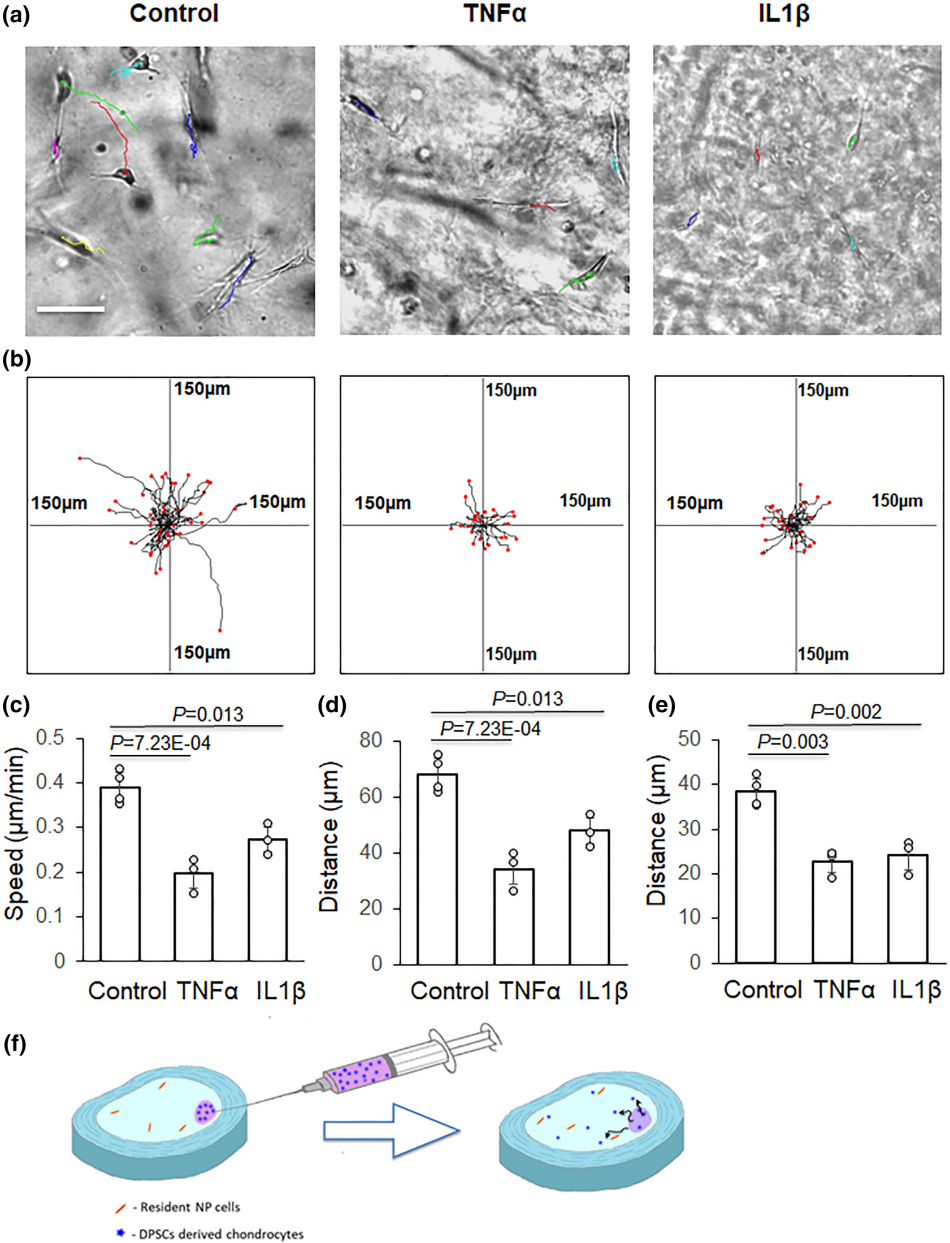
Quantitative results of migration of DPSC‐derived chondrogenic cells in collagen hydrogels loaded with IL‐1β or TNFα. (a) Cell migration pathways are labeled with lines. Scale bar: 100 μm. (b) Summary of migration pathways of cells from different experiments; center of each frame indicates migration initiation position of all cells under each experimental condition. (c) Cell migration speed. (d) Accumulated distance. (e) Euclidean distance. (f) Schematic diagrams showing the migration of cultured cells in collagen gel and the grafted cell migration after injection into the nucleus pulposus.

In this study, we observed the negative effect of IL‐1β and TNFα on differentiated cell migration in the hydrogels (Figure 4a). After the DPSC‐derived chondrogenic cells were grown in type I collagen hydrogels loaded with IL‐1β and TNFα, the cell migration velocities were 0.27 ± 0.03 μm/min and 0.20 ± 0.03 μm/min respectively, which are significantly lower than the velocity of cells in the control gel without IL‐1β and TNFα treatment (*p* < 0.01) (Figure 4c). The reduced cell migration speed led to the deceased cell migration distance (Figure 4d,e).

## 4 DISCUSSION

DPSCs are generally considered a type of MSCs. DPSC cells are easily accessible and have demonstrated similar differentiation capability as other MSCs. DPSC‐derived chondrogenic cells possess the capability of regenerating cartilage and the NP. In NP regeneration, cells could be injected into the nucleus tissue. To generate the regeneration function, the grafted cells may migrate from the injection site and spread into the nucleus tissue. In another approach, the cells can be delivered into the NP with collagen hydrogel. In this approach, the cells can be grown in the collagen hydrogel before the gel is injected into the lesion. Collagen hydrogel creates a permissive environment for grafted cell growth. In one clinical trial, a patient's autologous bone‐marrow stromal cells were isolated and expanded in a culture. The porcine tendon type I collagen gel encapsulating the cells was grafted to repair the articular cartilage defect in the medial femoral condyle. Results of that study show that a hyaline‐like type of cartilage tissue filled the cartilage defect, and the patient symptoms improved significantly (Kuroda et al., 2007).

A previous study showed that collagen coating in a cell culture plate increased MSC adhesion, proliferation, and migration when compared with a non‐treated tissue culture plate, and a fibronectin or poly‐L‐lysine (PLL)‐coated tissue culture plate (Somaiah et al., 2015). In another study, the migration of MSCs on an electrochemically aligned collagen (ELAC) bundle was observed (Gurkan et al., 2010). The migration of human MSCs (Xie et al., 2017) and rat bone marrow stromal cells (Hesse et al., 2010) was observed on type I collagen hydrogels. In that study, the cell migration velocity was different at early and late culture stages. The migration speed decreased after culturing for 2 weeks in either type I or type II collagen gel. The study indicates that the short‐time culturing of seeded cells in the collagen gel can maintain better cell motility after injection of the gel into the NP tissue, and the cells may have better capability of dispersing into the tissue.

The proliferation of human NP cells in type II collagen hydrogels was observed after the cells were cultured for 10 days (Priyadarshani, Li, Yang, & Yao, 2016). It is important to know if the collagen gels support the proliferation of DPSC‐derived chondrogenic cells. In this work, the alamarBlue® assay showed DPSC‐derived chondrogenic cells proliferated in both type I and type II collagen gels after culturing for 2 weeks. This observation indicates that the cell behavior transitions from higher motility at the early culture stage to lower motility after a longer period of cell culturing, and then the cells settle down and proliferate in the gels. Results indicate that cell motility is higher at an early culture stage, which may be the optimal time for cell transplantation.

MSC‐derived chondrogenic cells have demonstrated the capability to repair a cartilage defect (Liu et al., 2022; Pelttari et al., 2008; Solchaga et al., 2011). It is important to understand the difference in cell motility on the cartilage surface and inside the NP. To mimic cell migration under these two conditions, we studied the migration of cells on a type II collagen‐coated cell culture dish and in type II collagen gels. We found that the cell migration velocity is much higher on a stiff substrate such as the cell culture dish, compared with cells in the soft gels. Under both conditions, cell navigation was guided by the extended processes. Cells showed the ability to reverse their migration direction by switching the leading and trailing processes in the collagen gel or on the collagen‐coated surface. Our study also observed different cell morphologies in the gel and on the cell culture dish surface. The flattened cell shape on the dish surface indicates extensive cell attachment to this stiff surface. In collagen gels, the smaller cell body size may allow it to move through the porous structure of the collagen gel.

After the DPSC‐derived chondrogenic cells are grafted into degenerated NP or cartilage, the cells will be under the influence of chemokines, such as IL‐1β and TNFα. In one study, chondrogenic progenitor cells isolated from human cartilage demonstrated migratory behavior. After treated with IL‐1β (1 ng/mL) and TNFα (10 ng/mL), cell motility was reduced significantly (Joos et al., 2013). That study showed the negative effect of IL‐1β and TNFα on chondrogenic progenitor cell migration. However, how these chemokines affect the cell motility of DPSC‐derived chondrogenic cells has not yet been reported. In this study, to mimic the in vivo condition, similar levels of IL‐1β (2 ng/mL) and TNFα (20 ng/mL) as used in previous study (Joos et al., 2013) were incorporated into collagen hydrogel before the cells were seeded in it. Because the cells showed higher motility in the type I collagen hydrogel than the type II collagen hydrogel, the impact of IL‐1β and TNFα on cell migration can be clearly demonstrated in the type I collagen hydrogel. Additionally, the collagen gels containing the DPSC‐derived chondrogenic cells can be grafted into the degenerated NP and cartilage in the treatment. Based on these considerations, we evaluated the effect of IL‐1β and TNFα on cell migration in type I collagen gels. Results of our study indicated that motility decreased after the cells were grown in type I collagen hydrogel containing IL‐1β or TNFα compared with non‐treated gels. This study indicates that the control of IL‐1β and TNFα production when the cells are grafted may help cell migration in the matrix.

## AUTHOR CONTRIBUTIONS

L. Y., Conceived and designed research; N. F, P. K, L. Y., performed experiments; L. Y., N. F, P. K., analyzed data; L. Y., N. F, P. K., interpreted results of experiments; L. Y., N. F., prepared figures; L. Y., drafted manuscript; L. Y., edited and revised manuscript; L. Y., N. F, P. K., approved final version of manuscript.

## FUNDING INFORMATION

This work was supported by the graduate student fellowship of Regional Institute of Aging and Kansas Idea Network of Biomedical Research Excellence (K‐INBRE) of National Institute of General Medical Sciences (P20GM10341).

## CONFLICT OF INTEREST STATEMENT

No conflicts of interest, financial or otherwise, are declared by the authors.

## ETHICS STATEMENT

No animal or human studies are involved. Ethical statement is not applicable.

## Supporting information


**Video S1.** Cell migration on type II collagen‐coated dish.

**Video S2.** Cell migration and migration direction change on type II collagen‐coated dish.

**Video S3.** Cell migration in type II collagen hydrogel.

**Video S4.** Cell migration and migration direction change in type II collagen hydrogel.

## Data Availability

The data that support the findings of this study are available upon reasonable request.
